# The Association Between Family Planning and Psychological Distress in Individuals With Minoritized Gender Identities in Germany: A Cross‐Sectional Study During the COVID‐19 Pandemic

**DOI:** 10.1111/psrh.70038

**Published:** 2025-11-05

**Authors:** Falk Batz, Alaleh Zati Zehni, Eva Lermer, Maximilian Berger, Lennard Schröder, Joachim Behr, Sven Mahner, Christian J. Thaler, Pichit Buspavanich

**Affiliations:** ^1^ Department of Obstetrics and Gynecology and Center for Gynecological Endocrinology and Reproductive Medicine University Hospital, Ludwig‐Maximilian‐Universität München Munich Germany; ^2^ Research Unit Gender in Medicine, Charité ‐ Universitätsmedizin Berlin Corporate Member of Freie Universität Berlin and Humboldt‐Universität Zu Berlin Berlin Germany; ^3^ Kinderwunsch Centrum München Munich Germany; ^4^ Department of Business Psychology Technical University of Applied Sciences Augsburg Augsburg Germany; ^5^ Center for Leadership and People Management Ludwig‐Maximilian‐Universität München Munich Germany; ^6^ Department of Psychiatry and Neuroscience, Charité ‐ Universitätsmedizin Berlin Corporate Member of Freie Universität Berlin and Humboldt‐Universität Zu Berlin Berlin Germany; ^7^ Department of Psychiatry, Psychotherapy and Psychosomatics Brandenburg Medical School Theodor Fontane Neuruppin Germany; ^8^ Faculty of Health Sciences Brandenburg, Joint Faculty of the University of Potsdam Brandenburg University of Technology Cottbus‐Senftenberg and Brandenburg Medical School Potsdam Germany; ^9^ Institute of Sexology and Sexual Medicine Charité ‐ Universitätsmedizin Berlin, Corporate Member of Freie Universität Berlin and Humboldt‐Universität Zu Berlin Berlin Germany; ^10^ German Center for Mental Health (DZPG), partner Site Berlin/Potsdam Berlin Germany

**Keywords:** desire for parenthood, gender identities, psychological distress

## Abstract

**Background:**

Among transgender and gender diverse (TGD) individuals there is an increasing desire for parenthood. TGD individuals must overcome unique legal, social and physiological obstacles to realize their desire to become parents. Indeed, since the onset of the COVID‐19 pandemic, TGD individuals have experienced a significantly higher prevalence of anxiety and depression, which may be strenuous to all areas of life, including family planning. The aim of this study was to evaluate the impact of the COVID‐19 pandemic in Germany on decisions regarding family formation in TGD compared to cisgender individuals, and what role psychological distress might play in this.

**Methods:**

The online survey included general demographic questions, domain‐specific questions including gender identity and sexual orientation, questions related to psychological distress, the desire for parenthood as well as motives for and against parenthood.

**Results:**

The desire for parenthood was lower and the level of depressive symptoms was higher in TGD (*n* = 187) than in cisgender individuals (*n* = 2135) during the COVID‐19 pandemic. Moreover, the desire for parenthood was associated with a lower level of depressive symptoms, younger age, having one or more children and living in an urban area. Further associations are higher scores in the desire for emotional stabilization, as well as lower scores in fear of personal constraints and the desire for social recognition.

**Conclusion:**

Our findings revealed that TGD individuals reported significantly higher levels of depressive symptoms compared to cisgender participants and expressed a lower desire for parenthood. These results highlight the need for targeted support from social services and health care providers, particularly in times of crisis such as the COVID‐19 pandemic. Tailored interventions are essential to address the mental health burden and to reduce the additional hurdles TGD individuals face when considering parenthood.

## 1 Introduction

The COVID‐19 pandemic has had a devastating impact on citizens and livelihoods worldwide, with negative social, psychological, and economic effects on individuals. As of May 2, 2023, there have been more than 170,000 deaths in Germany and more than 6.8 million deaths worldwide [1]. In addition to morbidity and mortality, the pandemic and its precautionary health measures have had enormous effects on physical and psychological well‐being, as illustrated by decreased general well‐being, impaired quality of life and increased degrees of psychological distress related to uncertainty, fear, and grief during the pandemic [2, 3]. These influences on psychological distress may lead to an increase in psychiatric disorders, traumatic stress, anxiety, and depression [4, 5]. Here, socially marginalized groups represent a particularly vulnerable group with disproportionately higher levels of health issues during the COVID‐19 pandemic [6, 7]. Compared to the rest of the population, communities that have already been marginalized by systemic discrimination and oppression are at higher risk of suffering from COVID‐19 [8].

The higher prevalence of psychological burden on transgender and gender diverse (TGD) individuals has been documented prior to the COVID‐19 pandemic. Thus, crises such as the COVID‐19 pandemic may affect this vulnerable group to a greater extent [9]. TGD individuals indicate significantly and disproportionately higher rates of depression and anxiety in comparison to cisgender individuals since the COVID‐19 pandemic [9, 10, 11]. Additionally, existing disparities in the level of psychological distress due to lower levels of social support, discrimination and internalized stigmatization in TGD individuals may negatively influence the susceptibility of TGD individuals to COVID‐19‐related psychological stress [12]. Social integration, such as having supportive families and friends or relationships has been proven to protect from potential psychological problems in TGD individuals and individuals with minoritized sexual identities [13, 14, 15, 16]. Yet, COVID‐19‐related confinement measures may limit access to those protective factors.

The pandemic also created a variety of triggers that mirrored and intensified the biographical challenges faced by TGD individuals. The widespread uncertainty surrounding the pandemic reflected the ambiguity and unpredictability encountered in external environments, such as societal acceptance and legal protection [17]. Social isolation measures disrupted access to affirming networks, exacerbating feelings of loneliness [18], while disruptions in healthcare systems delayed gender‐affirming surgeries and hormone therapies, exacerbating gender dysphoria [19]. Furthermore, social distancing measures restricted self‐expression, forcing some to remain in unsupportive or abusive households [18]. Fear of discrimination in healthcare settings deterred many TGD individuals from seeking COVID‐19‐related medical care, compounding stress and anxiety [20]. These factors contributed to worsened mental health outcomes, with increasing rates of depression and anxiety in TGD individuals compared to the general population [21]. Importantly, this does not suggest that TGD individuals are inherently more vulnerable. Instead, systemic factors such as discrimination, social marginalization, healthcare inequities, and economic instability amplified their susceptibility to external and internal stressors during the COVID‐19 pandemic. This perspective is consistent with the minority stress model, which posits that individuals from marginalized groups face chronic stressors due to stigma, prejudice, and discrimination. In the context of family planning, such stress processes may also affect whether and how TGD individuals pursue parenthood [22, 23].

Furthermore, social distancing measures and psychological distress during the COVID‐19 pandemic have fostered depressive symptoms and anxiety with impacts on couple stability, physical and emotional intimacy and proximity, sexual well‐being and the desire to start a family, regardless of gender and sexuality [24, 25, 26]. Previous studies have shown that the desire for parenthood can be positively associated with mental health, as an unfulfilled desire for parenthood is correlated with impaired mental well‐being and that the desire for parenthood can be a predictor for the development of depression [27, 28]. On the other hand, recent studies indicate that voluntary childlessness does not entail adverse health implications and that adults without children report similar levels of life satisfaction compared to parents and other non‐parents [29, 30].

Yet, current literature does not differentiate between gender identities and gender‐specific social, medical and legal obstacles individuals must overcome during the process of becoming a parent. Compared with cis‐heterosexual individuals, TGD individuals must overcome additional hurdles to become parents. First, TGD individuals have limited national legal possibilities of becoming parents: in certain jurisdictions, sterilization was a prerequisite for legal gender change among TGD individuals [31]. In Germany, the forced sterilization law for TGD individuals has only been dropped from the law in 2011. With forced neutering, TGD individuals lost fertility and were deprived of the possibility of becoming biological parents after transitioning [31, 32]. Furthermore, in the past some TGD individuals were legally denied access to their children after transitioning as some custody disputes presumed a potentially damaging effect on children [33, 34]. Today, studies revoke the potentially harmful effect on children of TGD parents and rather show positive outcomes for these children. With the changing of the legal situation and innovations in reproductive medicine, new options for TGD individuals to become parents have been created [35, 36].

Since COVID‐19, both cis‐heterosexual individuals and TGD individuals who plan to become parents face additional burdens and worries that are unique to the pandemic. For example, the restrictive access to reproductive treatment or worries due to the lack of scientific evaluation of COVID‐19‐related effects on pregnancy [26]. However, most existing research on family formation has focused primarily on cisgender individuals, making the experiences and parenthood desires of TGD populations less well understood. Research on family formation has focused on cisgender individuals. The literature on the desire for parenthood in TGD individuals reports heterogeneous findings: While motives for and against having children do not differ between cisgender and TGD individuals [37, 38, 39], TGD individuals tend to think longer about the motives and meaning of their desire to become parents [38, 40]. Findings from Kleinert et al. 2015 show no differences in the motivation for or against parenthood between TGD and cisgender individuals. The authors concluded that specific conditions of TGD individuals, such as discrimination and internalized stigmatization, have no (or very little) influence on the decision of whether to become a parent or not [37]. However, a recently published review reported a lower desire to become parents in TGD compared to cisgender individuals [41].

### 1.1 Aim of the Present Study

The aim of this study was to evaluate the impact of the COVID‐19 pandemic and the confinement measures in Germany on decisions regarding the desire for parenthood in TGD compared to cisgender individuals, and to determine what role psychological distress might play in this impact. Since general well‐being decreased significantly in TGD compared to cisgender individuals since the COVID‐19 pandemic [9], First, we want to examine if TGD individuals indicate higher levels of depressive symptoms in comparison to cisgender individuals (Research Question: RQ 1). Secondly, we aim to explore change in the desire for parenthood since the COVID‐19 pandemic (Research Question: RQ 2). Next, we want to examine whether there is a difference in the prevalence of desire for parenthood in TGD compared to cisgender individuals since the COVID‐19 pandemic (Research Question: RQ 3). Furthermore, we aimed to explore the motives for and against the desire for parenthood in cisgender compared to TGD individuals during the COVID‐19 pandemic (Research Question: RQ 4). Moreover, we examined the impact of age, residential environment, relationship status, parenthood, depressive symptoms, motives for or against having children in association with the desire for parenthood among TGD and cisgender individuals (Research Question: RQ 5).

## 2 Materials and Methods

### 2.1 Setting, Study Design and Sample

We generated an anonymous, nationwide, online cross‐sectional, open‐access survey using SoSci Survey Version 3.2.23 and made it available to users. We developed items for sociodemographic data. Additionally, we used standardized, validated instruments such as the four‐item Patient Health Questionnaire (PHQ‐4) and the Leipzig Questionnaire on Motives for Having a Child (LKM‐20). To maximize participation, we kept the inclusion criteria broad, requiring only (1) a minimum age of 18 years and (2) knowledge of German.

Between April 20 and July 20, 2020, we administered the survey in German and distributed the link via social media and online platforms. We shared invitations through Facebook, Instagram, X (Twitter), and WhatsApp, including public discussion forums (e.g., student exchange platforms, hobby‐related interest groups) and city‐ or region‐specific bulletin boards (e.g., “Bulletin Board Hamburg”). We also promoted the survey in LGBTQIA support groups such as “Queer in Germany (LGBTQ+)” and within networks of LGBTQIA organizations, including the Trans‐Ident Association in Germany (Trans‐Ident e.V.). In addition, we disseminated the survey through websites and magazines targeting gender minorities and via email distribution lists of Ludwig Maximilian University of Munich. Some participants promoted the survey within their own networks (snowball sampling). Prior to data collection, all participants reviewed and accepted an online‐based consent page that included information on the research project. Participation in the study was anonymous, voluntary and was not compensated. The Ethics Review Committee of the Faculty of Medicine, LMU Munich registered the survey (registration number: 20–344 KB) and conducted it in accordance with the Declaration of Helsinki.

### 2.2 Measures

#### 2.2.1 Gender Identity

Gender identity and sexual orientation were assessed with the item “In your opinion, which of the following categories most apply to you?”. The following answer categories were provided: *female, male, cis* (“I identify with the gender assigned at birth”), trans* (“I do not identify with the gender assigned at birth”) and *others*. Multiple answers were possible. For the analysis, participants were classified into six groups based on their self‐assigned gender identity: (I) cis‐women, (II) cis‐men, (III) trans* women, (IV) trans* men, (V) non‐binary gender and identities (participants who do not identify as female and male). The star (*) indicates that the respective terms include further gender identities beyond the expressions transgender and gender non‐conforming, respectively. The two analytical groups of this study are: cisgender individuals (cis‐women and cis‐men) and TGD individuals (trans* women, trans* men and non‐binary gender identities). For the multivariate analysis we used the dichotomous variable *gender identity* (TGD versus cisgender individuals).

#### 2.2.2 Desire for Parenthood

Further the respondents were asked if they had children (*yes, no*) and if they had the desire for children (*yes, no*).

#### 2.2.3 Symptoms of Depression

We used the PHQ‐4, a standardized brief self‐report questionnaire to measure current symptoms of depression and anxiety [42]. The PHQ‐4 is an instrument used to measure symptom load, level of functional impairment, and possible disability in everyday life [43]. The PHQ–4 does not serve as a diagnostic tool, but rather serves as a screening tool for the indication of further examination to identify potential cases of depression and anxiety and to ensure a sufficient treatment of mental disorders [43]. The four items assess the subjective psychological level of clinically significant depression over a 14‐day period (“Feeling nervous, anxious, or on edge”; “Not being able to stop or control worrying”, “Feeling down, depressed, or hopeless”; “Little interest or pleasure in doing things”). Responses are scored from (0) “Not at all”, [2] “*Several days*”, [24] “More than half the days” or [12] “Nearly every day”; range = 0 to 12). Higher values indicate higher scores of depressive symptoms. A (PHQ–4 total score of 3 to 5 indicates mild, a score of 6 to 8 moderate, and a sum score of 9 or more represents a severe elevation [43].

#### 2.2.4 Motives for and Against Having Children

The motives for and against own parenthood were assessed by means of the LKM‐20 [44]. The LKM‐20 is a 20‐items‐questionnaire to investigate motives in favor and against having children. Participants were asked to what extend a certain motive influences their personal decisions for or against having children. Their attitudes towards various motives for their own parenthood were assessed as followed: (1) *Does not influence me at all*, (2) *Does moderately influence me*, (3) *Does partially influence me*, (4) *Does reasonably influence me*, (5) *Does strongly influences me*. The items form four LKM‐20 subscales with a sum‐score of each scale, which are characterized as follows: Scale 1. The desire for emotional stability and life meaning. Sample item: “A child gives me the feeling of having a real home*”*. Scale 2. Fear of personal constraints. Sample item: “With a child my partner and I do not have enough time for each other”. Scale 3. The desire for social recognition. Sample item: “A child is necessary for me to be acknowledged as an adult”. Scale 4. Fear of financial constraints. Sample item: “Having a child in our society is a financial burden*”*.

#### 2.2.5 Sociodemographic

Participants reported their age, which we categorized into three groups: 18 to 25 years, 26 to 35 years, and 36 years and older for analysis. To capture the residential environment, the participants indicated the size of their place of residence; we dichotomized the responses into urban areas with 20,000 or more inhabitants and rural areas with fewer than 20,000 inhabitants. We assessed relationship status by asking participants whether they were single or in a relationship, and this variable was included as a categorical covariate in the analyses.

### 2.3 Statistical Analyses

This study focuses on the impact of psychological distress during the COVID‐19 pandemic on family planning and motives for or against parenthood in TGD compared to cisgender individuals. Due to missing values, 131 participants were excluded from the analysis. Further due to the low number of inter* participants we did not include this group in the current study. Therefore, eligible participants for analysis resulted in *N* = 2322 participants.

First, descriptive statistics were calculated for all subgroups (I) cis‐women, (II) cis‐men, (III) trans* women, (IV) trans* men, (V) non‐binary gender identities and the variables of interest (Tables 1, 2). A two‐sample t‐test was used to compare the groups' cisgender versus TGD participants. An ANOVA with posthoc test (LSD) was conducted to gain more differentiated insights (RQ 1). Furthermore, to examine whether there is a difference in the prevalence of desire for parenthood in cisgender compared to TGD participants during the pandemic a chi‐square test was conducted (RQ 2). Table 2 shows descriptive information on changes in the desire for parenthood since the COVID‐19 pandemic (RQ 3).

**TABLE 1:** Descriptives of cisgender versus TGD individuals.

All participants	Cisgender individuals		TGD individuals		
*N* = 2322	Women *n* = 1640	Men *n* = 495	Trans* women *n* = 60	Trans* men *n* = 96	Non‐binary *n* = 31
Age					
18–25 years 805	588 (35,9)	169 (34.8)	12 (20.0)	27 (28.1)	9 (30.0)
26–35 years 1000	731 (44.6)	194 (40.0)	34 (56.7)	30 (31.3)	11 (36.7)
36–45 years 383	255 (15.6)	72 (14.8)	12 (20.0)	36 (37.5)	8 (26.7)
Over 46 years 121	64 (3.9)	50 (10.3)	2 (3.3)	3 (3.1)	2 (6.7)
Relationship status					
Single 753	467 (28.5)	184 (37.2)	28 (46.7)	58 (60.4)	16 (51.6)
In a relationship 1569	1173 (71.5)	311 (62.8)	32 (53.3)	38 (39.6)	15 (48.4)
Residential environment					
Metropolis^a^ 1468	1023 (62.5)	315 (63.6)	46 (76.7)	59 (61.5)	25 (80.6)
Medium sized town^b^ 335	244 (14.9)	65 (13.1)	10 (16.7)	12 (12.5)	4 (12.9)
Small town^c^ 284	185 (11.3)	73 (14.7)	3 (5,0)	21 (21.5)	2 (6.5)
Rural community^d^ 231	184 (11.2)	42 (8.5)	1 (1.7)	4 (4.2)	0 (0.0)
Employment status					
Self‐employed 127	95 (5.8)	21 (4.3)	4 (6.7)	5 (5.3)	2 (6.5)
Employed 1225	851 (51.9)	267 (54.2)	38 (63.3)	52 (54.7)	17 (54.8)
Student 793	580 (35.4)	181 (36.7)	7 (11.7)	18 (18.9)	7 (22.6)
Not employed 171	111 (6.8)	24 (4.9)	11 (18.3)	20 (21.1)	5 (16.1)
COVID‐19 status					
Infected, symptoms 3	0 (0.0)	3 (0.6)	0 (0.0)	0 (0.0)	0 (0.0)
Infected, no symptoms 5	3 (0.2)	2 (0.4)	0 (0.0)	0 (0.0)	0 (0.0)
Previous infected 13	9 (0.6)	4 (0.8)	0 (0.0)	0 (0.0)	0 (0.0)
Not infected/not tested 2257	1599 (99.3)	474 (98.1)	58 (100)	96 (100)	30 (100)
Clinically significant depression (PHQ‐4^e^)					
No 1509	1114 (77.4)	311 (73.5)	23 (42.6)	46 (59.7)	15 (51.7)
Yes 514	326 (22.6)	112 (26.5)	31 (57.4)	31 (40.3)	14 (48.3)

Abbreviation: TGD, transgender and gender diverse.

^a^ 100.000 or more inhabitants.

^b^ 20.000 to 100.000 inhabitants.

^c^ 5.000 to 20.000 inhabitants.

^d^ Up to 5.000 inhabitants.

^e^ Depressive symptoms measured by PHQ‐4 score: A total raw score ≥ 6 indicates clinically significant depression and/or anxiety.

* The star (*) following trans* indicates that the term also includes further gender identities beyond transgender, transident or transsexual.

**TABLE 2:** Parental status and family planning.

*N* = 2322	Cisgender individuals		TGD individuals		
	Women *n* = 1640	Men *n* = 495	Trans* women *n* = 60	Trans* men *n* = 96	Non‐binary *n* = 31
Current Parenthood					
No	1299 (84.8)	359 (80.1)	52 (86.7)	82 (88.2)	21 (84.0)
Yes	233 (15.2)	89 (19.9)	8 (13.3)	11 (11.8)	4 (16.0)
Desire for parenthood					
No	245 (16.0)	68 (15.2)	16 (26.7)	37 (41.1)	8 (25.8)
Yes	1284 (83.9)	378 (84.8)	44 (73.3)	53 (58.9)	23 (74.2)
Desire for parenthood since the COVID‐19 pandemic					
Increased	133 (8.8)	59 (13.3)	15 (25.4)	19 (20.2)	9 (29.0)
Decreased	140 (9.2)	23 (5.2)	6 (10.2)	6 (6.4)	5 (16.1)
Did not change	1245 (82.0)	362 (81.5)	38 (64.4)	69 (73.4)	17 (54.8)

*Note: p* < 0.05 was considered statistically significant.

Abbreviation: TGD, transgender and gender diverse.

* The star (*) following trans* indicates that the term also includes further gender identities beyond transgender, transident or transsexual.

To compare motives for and against having a child in cisgender and TGD individuals during the COVID‐19 pandemic, we used two‐sample t‐tests (RQ 4). To investigate RQ 5, we tested whether there was an association between the gender identities (cisgender versus TGD individuals) and the current motives for and against having a child by performing a multivariate logistic regression model, whereby the desire for parenthood was the dichotomous, dependent variable. We included the following covariates in the model: gender identity (cisgender versus TGD individuals), age (18–25 years, 26–35 years versus 36 years and above), residential environment (urban cities versus rural communities under 20,000 inhabitants), relationship status (single versus in a relationship), parenthood (yes versus no), depressive symptoms (PHQ‐4 total score), motives for and against the desire for parenthood (sum‐scores of each LKM‐20 scale).

We performed basic assumptions for logistic regression including independence of errors, linearity in the logit for continuous variables, absence of multicollinearity, and lack of strongly influential outliers which were suitable for our analysis. To verify the normal distribution of the sample for metric variables we used the Kolmogorov–Smirnov test. In the present study, the scale of the PHQ‐4 showed very good internal consistency (Cronbach's alpha = 0.848). We set the level of statistical significance at *p* < 0.05 (two‐tailed) for all analyses. Statistical analysis was performed with SPSS version 26 (IBM Corp. Released 2019. IBM SPSS Statistics for Windows, Version 26.0. Armonk, NY).

## 3 Results

In total, *N* = 2322 participants were included in the analysis (Table 1). Of those, *n* = 2135 (91.9%) self‐identified as cisgender individuals and *n* = 187 (8.1%) as TGD individuals. Concerning age, most participants were younger than 36 years old (*n* = 1805, 78.2%, TGD individuals: *n* = 123 and cisgender individuals *n* = 1682). Regarding the residential environment, most of the participants *n* = 1803 (77.6%) lived in urban cities with more than 20,000 inhabitants. Most of the participants across all groups *n* = 1352 (58.2%) were currently working and a total of *n* = 793 (34.2%) participants were students. Only *n* = 8 participants reported a current COVID‐19 infection and *n* = 13 reported a previous COVID‐19 infection. In terms of the prevalence of clinically relevant depressive symptoms, relatively more TGD individuals (*n* = 76; 47.5%) indicated moderate to severe depressive symptoms in comparison to cisgender individuals (*n* = 438; 23.5%). The highest level of depressive symptoms was reported in trans* women (*n* = 31; 57.4%) and non‐binary individuals (*n* = 14; 48.3%).

To investigate RQ 1 a two‐sample t‐test revealed that participants being part of the cis‐gender group (M = 3.68, SD = 2.70) had a significantly lower mean level of depressive symptoms compared to TGD individuals (M = 5.00, SD = 3.23, t (2.26) = −6.28, *p* < 0.001, d = −0.443). ANOVA with posthoc test (LSD) results showed a significant group effect (F = 15.46, *p* < 0.001) and revealed that trans* women (*p* < 0.001, MD = −1.85), trans* men (*p* < 0.05, MD = −0.80) and non‐binary individuals (*p* < 0.001, MD = −1.90) showed significantly higher levels in depressive symptoms compared to participants from the cisgender group. For more details, see the descriptive statistics in Table 1.

### 3.1 Parental Status and Family Planning

Most participants (*n* = 1813, 84.0%) reported having no children (cisgender individuals *n* = 1658; TGD individuals *n* = 155). Among all the participants, a total of 1782 individuals (82.7%) reported a desire for parenthood. Upon further examination (RQ 2) of individuals' desire for parenthood within gender identity groups, 66.3% of TGD individuals (*n* = 120) indicated a desire, compared to 84.2% of cisgender individuals (*n* = 1662). Of all participants, 180 individuals (8.4%) reported a decrease in their desire for parenthood, while 235 individuals (11.0%) reported an increase. The majority, 1731 participants (80.7%), reported no change in their desire to become parents during the COVID‐19 pandemic (RQ 3). For more details see Table 2.

### 3.2 Motives for and Against Having Children (LKM‐20)

Table 3 presents an overview of the motives for and against having children during the COVID‐19 pandemic (RQ 4). For both subgroups, cisgender individuals as well as TGD individuals, the hierarchy of motives for and against the desire for children since the COVID‐19 pandemic was similar [45]. From all areas of the LKM‐20, the motive “Desire for emotional stability and life meaning” (Scale 1) had the greatest influence on the desire for own parenthood [37]. This motive was followed by Scale 4: “Fear of financial constraints”. Scale 2 “Fear of personal constraint” appeared substantially less significant. Subsequently Scale 3 “Desire for social recognition” showed the least influence on the desire for parenthood. Furthermore, a binary logistic regression model (RQ 5; Table 4) with *Desire for having children* as a dependent variable revealed that lower levels of depressive symptoms and a younger age, living in an urban area, and having one or more children were significantly associated with the desire for parenthood. Cisgender individuals have about a 2.5 higher probability of having a desire to become a parent than TGD individuals. Concerning motivation for having children, higher scores of *Desire for emotional stabilization* (LKM‐20 Scale 1) and lower scores of *Fear of personal constraints* (LKM‐20 Scale 2) and *Desire for social recognition* (LKM‐20 Scale 3) were significantly associated with the desire for parenthood. The relationship status and *Fear of financial constraints* (LKM‐20 Scale 4) had no significant effect.

**TABLE 3:** Comparison of Motives for and against the desire for parenthood in cisgender individuals and TGD individuals since the COVID‐19 pandemic.

LKM‐20 scales	Cisgender individuals			TGD individuals				
	1537	2.92	1.31	163	2.81	1.53	0.33	0.077
(1) Desire for emotional stabilization	1537	2.92	1.31	163	2.81	1.53	0.33	0.077
(2) Fear of personal constraints	1540	2.03	0.85	163	2.19	1.33	0.03	−0.143
(3) Desire for social recognition	1555	1.49	0.79	165	1.68	1.29	0.01	−0.178
(4) Fear of financial constraints	1547	2.43	0.98	164	2.66	1.34	0.07	−0.196

*Note: p* < 0.05 was considered statistically significant.

Abbreviations: d, Cohen's d; LKM, Leipzig questionnaire on motives for having a child; M, mean; *p*, *p* value; SD, standard deviation; TGD, transgender and gender diverse.

**TABLE 4:** Logistic Regression model with “Desire for parenthood” as the dependent variable.

	Regression coefficient	Odds‐ratio	Standard error	*p*	95% confidence interval lower upper	
Gender identity						
TGD^a^ individuals (reference)	—	—	—	—		—
Cisgender individuals	0.953	2.593	0.264	**< 0.001**	1.546	4.351
Age group				**< 0.001**		
18–25 years	1.177	3.245	0.240	**< 0.001**	2.029	5.191
26–35 years	0.892	2.439	0.224	**< 0.001**	1.572	3.783
Over 35 years	—	—	—	—	—	—
Relationship status^b^	0.097	1.102	0.170	0.567	0.789	1.539
Residential environment^c^	−0.509	0.601	0.187	**0.006**	0.417	0.876
Parenthood^d^	1.274	0.280	0.327	**< 0.001**	1.883	6.789
PHQ‐4 total score^e^	−0.065	0.937	0.031	**0.035**	0.882	0.995
LKM‐20^f^ subscale 1^g^	1.482	4.401	0.103	**< 0.001**	3.595	5.387
LKM‐20 subscale 2^h^	−0.347	0.707	0.130	**0.008**	0.548	0.912
LKM‐20 subscale 3^i^	−0.316	0.729	0.153	**0.039**	0.540	0.985
LKM‐20 subscale 4^j^	−0.053	0.949	0.117	0.653	0.754	1.194
Intercept	−2.062	0.127	0.400	**< 0.001**		
Statistics for linear regression						
Nagelkerke's *R* ^2^	0.481					
Standard error	0.063					

*Note: p* < 0.05 was considered statistically significant.

^a^ TGD, transgender and gender diverse.

^b^ 1 = single, 2 = in a relationship.

^c^ 1 = urban cities, 2 = rural communities under 20,000 inhabitants.

^d^ 1 = no children; 2 = one or more children.

^e^ Depressive symptoms were measured with the Patient Health Questionnaire 4 items (PHQ‐4) total raw score.

^f^ LKM, Leipzig questionnaire on motives for having a child.

^g^ 1 = Desire for emotional stabilization.

^h^ 1 = Fear of personal constraints.

^i^ 1 =Desire for social recognition.

^j^ 1 = Fear of financial constraints.

## 4 Discussion

The present study provides unique evidence on the COVID‐19 pandemic‐related impact of depressive symptoms on the desire for parenthood among cisgender individuals and TGD individuals.

COVID‐19‐related information on individuals with minoritized gender identities is scarce as TGD individuals are often ignored in electronic health recordings and public health surveillance [46]. This is concerning, since the COVID‐19 pandemic and its confinement measures led to an increase in insecurity with impaired psychological well‐being and rising prevalence rates of depression and anxiety, especially in social minorities such as TGD individuals. Our study revealed that TGD individuals reported significantly higher levels of depressive symptoms compared to cisgender participants, confirming previous evidence of disproportionate psychological burdens during the COVID‐19 pandemic [9, 21, 47, 48]. These findings align with studies documenting that social isolation disrupted access to affirming networks and that interruptions in gender‐affirming healthcare increased dysphoria [17, 18, 19]. In our sample, such increased psychological distress was reflected in the higher proportion of TGD participants with clinically relevant depressive symptoms (47.5% vs. 23.5% in cisgender individuals). The lower reported desire for parenthood among TGD participants should be interpreted in the context of the minority stress model: structural barriers such as discrimination, legal restrictions, and healthcare inequities may reduce the feasibility of parenthood, which in turn may shape reported desires. Thus, our findings do not indicate an inherent vulnerability of TGD individuals but highlight how the pandemic magnified preexisting inequities, which in turn may affect both mental health and family‐planning decisions [20, 43]. The preexisting greater mental health burden for TGD individuals compared to cisgender individuals in Germany has been reported prior to the pandemic [49]. Kasprowski et al. discovered in a study of 4511 individuals identified as gender and sexual minorities living in Germany that TGD individuals are at a three times greater risk for mental health‐related disparities, such as depression and burnout, when compared to the cisgender population. Prior to the pandemic, 39% of trans individuals were diagnosed with relevant anxiety disorders in comparison to 9% of cisgender individuals living in Germany [11]. The COVID‐19 pandemic even led to an increase in psychological disorders like loneliness, anxiety, depression and greater prevalence of post‐traumatic stress disorders (PTSD) in TGD individuals [50, 51]. For example, previous empirical work indicated that the level of loneliness increased with the beginning of the COVID‐19 pandemic: 31% of trans* individuals versus 7% of a cisgender population experienced loneliness [22]. Similarly, Gonzales and colleagues (2020) reported that 60% of 477 college students in the United States who identified as sexual or gender minorities reported experiencing psychological distress. Additionally, 45.7% of the study sample experienced clinically significant depression [20]. While these findings align with previous literature and provide support for RQ2, it is essential to acknowledge the potential limitations in interpreting subgroup data. Significantly higher levels of depressive symptoms were identified among all subgroups of TGD individuals within our sample. Upon closer examination of the subgroups in our study sample, TGD individuals presented the highest rates of depressive symptoms, with non‐binary participants presenting the second highest rates. Cisgender individuals presented the lowest levels of depressive symptoms. Moreover, studies on the effects of the pandemic on trans* health care documented reduced access to gender‐affirming resources (e.g., endocrine therapies, surgical interventions), a protective factor for mental and physical health in TGD individuals [50, 51, 52]. Further obstacles like social isolation measures additionally impair the physical and mental health of TGD individuals including postponements or cancellations of psychological counseling and medical gender‐affirming treatment as well as other services (e.g., facial hair removal) which may lead to an increase in depression [50].

In addition, according to the literature, parenthood desire is positively associated with psychological well‐being [27, 28]. In RQ 5, a reduction in the desire for parenthood is anticipated as a result of an increase in depressive symptoms due to the COVID‐19 pandemic, particularly in TGD individuals compared to cisgender individuals [9, 26, 53]. Our research is in line with previous findings that indicate a negative correlation between impaired psychological well‐being and depression, with the desire to become a parent in both cisgender and TGD individuals [23, 27, 28]. Looking at the birth rates in Germany since the pandemic, the German Federal Statistical Office documented an increase of 10% in March 2021 compared to March 2020. In 2021 Germany had the highest birth rates in 20 years. Thus, despite lower well‐being and higher levels of depression and anxiety in Germany, the desire for parenthood has even increased since the COVID‐19 pandemic. This phenomenon has previously been observed during life‐threatening occurrences such as the 2001 Gujarat earthquake in India and Hurricane Mitch in Nicaragua in 1998. Both events were accompanied by high rates of mortality but resulted in an increase in birth rates [54, 55]. The increasing birth rates might also be explained by worries that the SARS‐CoV‐2 virus may lead to infertility. Furthermore, confinement measures may positively affect couples' intimacy and thereby reinforce the desire to become parents. Previous studies on the impact of the COVID‐19 pandemic present mixed results. Micelli and colleagues [26] reported a trend toward decreased well‐being during quarantine, with lower well‐being significantly linked to less desire to start a family in heterosexual couples: 37.3% of Italian couples with an intention to become parents abandoned the planning owing to the feeling of instability and fear of future economic difficulties and potential negative consequences in the case of an infection during pregnancy [26]. The pandemic appears to have reinforced heteronormative ideals among many cisgender individuals, who often returned to traditional family roles during the crisis. However, research shows that structural factors, not individual choices or comfort, explain the increased caregiving and decreased workforce participation among women [56, 57]. These circumstances intensified preexisting gendered divisions of labor, which shaped how cisgender individuals navigated family life and work during the crisis [17, 19]. In contrast, TGD individuals demonstrated significant adaptability and resilience, potentially shaped by their lived experiences navigating societal ambiguity and systemic barriers [9, 10]. TGD individuals might reframe uncertainty as an opportunity for growth, forming online communities and mutual aid networks to counter isolation [18, 21]. Additionally, their rejection of traditional gender roles may have allowed them greater flexibility in redefining life goals, including family planning and parenthood, during the pandemic [38, 40]. The divergent coping strategies adopted by TGD and cisgender individuals in response to the crisis may thus be understood not only as individual adaptations but also as outcomes influenced by broader societal norms and structural inequities [22, 23].

In our study, 66.3% of TGD individuals indicated a desire for parenthood compared to 84.2% of cisgender individuals. Furthermore, our findings indicated that cisgender individuals have about 2.5 times higher probability of having a desire for parenthood than TGD individuals when simultaneously adjusting for age, previous parenthood, residential environment relationship status and motives for and against the desire for parenthood. This finding is in line with previous studies showing a lower desire for parenthood in TGD individuals [58, 59]. In a Belgian study 27 out of 50 trans* men desired to become parents. The lower prevalence of desire for parenthood in TGD compared to cisgender individuals might be explained by the fact that there are more obstacles to becoming parents for TGD individuals: external factors such as legal constraints and social acceptance, and internal factors such as internalized stigmatization, may play a role in individuals' mental health. These cause additional hindrances with which cis‐heterosexual couples do not have to contend [60, 61]. In some jurisdictions sterilization was a necessary precondition to legally change gender [31]. Until 2011, Germany had a law forcing the sterilization of TGD individuals, making it impossible for them to have biological children after their transition [31, 32]. However, recent research has shown positive outcomes for children of TGD individuals, challenging these assumptions [35, 36]. With the changing of the legal situation and innovations in assisted reproductive techniques (ART), like Intracytoplasmic Sperm Injection (ICSI) and other methods such as heterologous sperm donation, new options to have biological children or even to become a parent to a non‐biological child have been created for TGD individuals. Furthermore, adoption, foster‐parenting and other family concepts like co‐parenting and step‐parenting offer additional possibilities for TGD individuals to become parents [31]. The current literature shows an increase in desire for parenthood in TGD individuals [31, 32, 59]. However, TGD individuals may, still encounter prejudices towards them compounded by a social mistrust of TGD individuals having children. TGD individuals may face internalized stigmatization and discrimination which can hinder them from realizing their desire to create a family [62].

The findings of the study indicated that the majority of the participants did not report a change in their desire for parenthood in both subgroups of TGD and cisgender individuals since the onset of the pandemic when asked retrospectively. The unique changes caused by the pandemic and social restrictions may not considerably influence family planning. However, it is imperative to acknowledge the limitations of the study's design, particularly its cross‐sectional nature and the reliance on self‐reported data, which may have introduced bias.

In terms of motives that influence the desire for parenthood, our study indicates that a higher score in “Desire for emotional stabilization” and a lower score in “Desire for social recognition” as well as the “Fear of personal constraints” were significantly associated with the desire for parenthood. These variables can be considered as “states” following the concept of “Trait and states” as they can influence the desire for parenthood [63]. Our findings align with previous research, which suggests that emotional motives play a significant role in the desire to become a parent for both sexual orientation and gender identity groups. However, the financial situation was reported as the most important external factor influencing the realization of parenthood desire in both groups.

Furthermore, it is important to mention that the state of not having children should be seen as a voluntary–involuntary continuum [64]: Childlessness should not be framed as a deficit but rather understood along a voluntary–involuntary continuum, reflecting diverse personal and structural circumstances. Thus, future studies should integrate this continuum when investigating influences on the desire for parenthood. Our results revealed that lower levels of depressive symptoms, a younger age, living in an urban area and having one or more children were significantly associated with the desire for parenthood. The literature on this subject is inconclusive. Umberson et al. [65] examined the literature concerning the desire for parenthood and discovered that becoming a parent during young adulthood was negatively correlated with psychological well‐being. This was primarily due to elevated stress levels as a result of economic insecurity and relationship issues [65]. Subsequently a reduction in the desire for further children may follow. In contrast, Kohler et al. described an association between having the first child and higher levels of well‐being, whereas having additional children was correlated with a decrease in well‐being [66]. Nevertheless, the desire for parenthood was described as protective for future psychological well‐being and negatively correlated to depression [28].

This study has substantial strengths: the national sample size is comparatively large with a substantial participation rate of TGD individuals. Furthermore, this is the first study which examined the desire for parenthood among TGD individuals compared to cis‐individuals during the COVID‐19 pandemic. In terms of age, most of the respondents were 35 years old or younger (78.2%). Thereby, the study sample reflects the most common age range during which the most German cis women attempt to become pregnant, according to the German Federal Statistical Office. The average age at which a woman in Germany gives birth to her first child is 30.2 years. The mean paternal age in Germany is 33.2 years [67]. With respect to the limiting factors of our study, the overrepresentation of 18‐ to 35‐year‐old participants might also be a limitation. Most previous studies on TGD individuals reported study samples of cisgender individuals who were older than the samples of TGD individuals. Consequently, the comparability of the results is limited. The PHQ‐4 used in this study for depression and anxiety screening is based solely on self‐reports and, therefore, cannot provide a diagnosis. Grobe and colleagues [68] reported that self‐report questionnaires generally detect fewer instances of psychological disorders, such as depression, than psychological or medical assessments [68]. Future research should include semi‐structured interviews to increase the objectivity of the research. Furthermore, we do not have longitudinal data of the desire for parenthood. It would have been interesting to follow our study sample of cisgender individuals and TGD individuals into the phase after the COVID‐19 pandemic to evaluate the desire to become a parent throughout the course of the pandemic and to determine which individuals indeed became parents. Lastly, this study has examined the desire to become a parent using a limited dichotomous variable in the multivariate analysis, which may overlook the complexities, nuances, and temporary fluctuations in individuals' varying degrees of parental desire. In order to gain a better understanding of the role of family planning in TGD individuals, future research should instead look at parenthood aspiration [69], as it includes not only the desire to become parents but also parenthood expectations and parenthood intentions. Follow‐up studies should further examine potential uncertainty or ambivalence about the desire for parenthood and add variables on the temporality of attitudes towards own parenthood.

## 5 Conclusion

This is the first study to examine the psychological distress of TGD individuals and cis‐individuals in light of their family plans in Germany during the COVID‐19 pandemic. Our results reveal higher levels of depressive symptoms among individuals identifying as gender minorities during the COVID pandemic in Germany. The desire for parenthood was associated with lower levels of depressive symptoms, a younger age, living in an urban area, having one or more children, higher scores in desire for emotional stabilization as well as lower scores in fear of personal constraints and desire for social recognition. Our results emphasize the requirement for social, psychological, and medical assistance during this pandemic regarding family planning and the desire to become parents.

Additional research is necessary to determine the extent to which an unfulfilled desire for parenthood affects mental and physical health during global pandemics. This will be especially critical for supporting vulnerable populations, including TGD individuals. To increase access to mental healthcare and resources such as peer support groups within queer communities, it is essential to expand services such as telemedicine that can reduce barriers to care during a pandemic. It is also important to consider close collaboration between primary and mental healthcare systems, as well as queer communities, to implement recommendations from clinical guidelines and improve daily routines.

## Ethics Statement

All human subjects provided written informed consent with guarantees of confidentiality. The survey was submitted to the Ethics Review Committee of the Faculty of Medicine, LMU Munich for consultation (registration number: 20–344 KB).

## Consent

Our research was carried out in accordance with the Declaration of Helsinki of the World Medical Association and informed consent was obtained from all participants.

## Conflicts of Interest

S.M. reports research support, advisory board, honoraria and travel expenses from AbbVie, AstraZeneca, Clovis, Eisai, GlaxoSmithKline, Medac, MSD, Novartis, Olympus, PharmaMar, Pfizer, Roche, Sensor Kinesis, Teva, and Tesaro. All other authors have no conflicts of interest to declare.

## Data Availability

Data is stored in a non‐public repository due to the sensitive nature of the data collected. Data is available from the corresponding author on request.
